# Case Report: Cytoreductive Surgery and HIPEC Associated With Liver Electrochemotherapy in a Cholangiocarcinoma Patient With Peritoneal Carcinomatosis and Liver Metastasis Case Report

**DOI:** 10.3389/fsurg.2021.624817

**Published:** 2021-03-19

**Authors:** Mauro Stefano, Enrico Prosperi, Paola Fugazzola, Beatrice Benini, Marcello Bisulli, Federico Coccolini, Costantino Mastronardi, Alessandro Palladino, Matteo Tomasoni, Vanni Agnoletti, Emanuela Giampalma, Luca Ansaloni

**Affiliations:** ^1^General and Emergency Surgery Department, Azienda Unità Sanitaria Locale Romagna Trauma Center “Maurizio Bufalini” Hospital, Cesena, Italy; ^2^Anesthesia and Intensive Care Department, Azienda Unità Sanitaria Locale Romagna Trauma Center “Maurizio Bufalini” Hospital, Cesena, Italy; ^3^Interventional Radiology Department, Azienda Unità Sanitaria Locale Romagna Trauma Center “Maurizio Bufalini” Hospital, Cesena, Italy; ^4^General Emergency and Trauma Surgery Department, Pisa University Hospital, Pisa, Italy

**Keywords:** heated intraperitoneal chemotherapy, cholangiocarcinoma, debulking, peritoneal metastases, electrochemotherapy

## Abstract

**Introduction:** Cholangiocarcinoma (CCA) is the second most common primary tumor of the liver, and the recurrence after hepatic resection (HR), the only curative therapy, is linked with a worse prognosis. Systemic chemotherapy (SC) and liver loco-regional treatments, like trans-arterial chemoembolization (TACE) or radio embolization (TARE), have been employed for the treatment of unresectable intrahepatic metastasis (IM) with benefit on overall survival (OS), but SC has a limited effect on peritoneal metastasis (PM). In the last years, novel treatments like electrochemotherapy (ECT) with bleomycine (BLM) for IM and cytoreductive surgery with hyperthermic intraperitoneal chemotherapy (CRS and HIPEC) for PM have been applied in small series but with encouraging results. We hereby describe the first synchronous application of ECT and CRS and HIPEC for the treatment of a patient with IM and PM from CCA.

**Case Description:** A 47-year-old male patient with CCA underwent HR followed by adjuvant SC. After 14 months, for the occurrence of IM, the patient underwent a second HR and SC. Nonetheless, a new recurrence occurred and a third attempt of HR was proposed. Due to the intraoperative finding of unresectable IM with PM, no resective procedure was performed and the patient was referred to our center. CRS and HIPEC with cisplatin and mitomycin for PM and ECT with BLM on a bulky metastasis of the hepatic hilum were performed after 38 months from the first HR. The length of hospital stay was 19 days. At the computed tomography (CT) performed 11 days after treatment complete necrosis of the treated IM was detected.

**Results:** CT scan after 3 and 6 months and magnetic resonance after 9 months were performed. Necrosis of the treated IM nor PM but progression of the residual liver lesions was observed. After 3 months, the patient received SC and underwent TACE after 8 months and TARE after 9 months for the residual liver metastases. At 14 months from CRS and HIPEC, the patient is alive, in good condition, and with stability of the disease.

**Conclusions:** The association of ECT and CRS and HIPEC could be safe and effective for the treatment of unresectable recurrent intrahepatic CCA with PM.

## Introduction

Cholangiocarcinoma (CCA) is the second most common neoplasia of the liver after hepatocellular carcinoma accounting for the 3–5% of all the gastrointestinal cancer (1). Because CCA is a silent neoplasia, from 10 to 20% of the patients have already developed peritoneal metastasis (PM) at the first diagnosis, precluding the radical surgical resection's attempt, which remains the only potentially curative treatment for the liver-confined disease (2). In particular, intrahepatic CCA is an highly lethal disease with 5-year survival rates of 23–42% vs. 0%, respectively following R0 and R+ resection, and 5-year survival rates of 0–9% and up to 43%, respectively, in patients with N1 and N0 status following surgical resection (3). Although in intrahepatic CCA patients the median overall survival is 15–40 months (4), in the presence of PM, the treatment remains palliative with the application of systemic chemotherapy (SC) with the overall survival dropping to a mean of 8.1 months (5) and to 14.5 months with the addition of hepatic artery based therapies (6).

Cytoreductive surgery (CRS) and hyperthermic intraperitoneal chemotherapy (HIPEC) has become over years an effective strategy for the treatment of cancers with PM, initially in the treatment of primitive peritoneal tumors [mesothelioma (7) and pseudomyxoma peritonei (8)], later on, the advanced stages of ovarian cancer (9), and more recently has been applied in selected center even for the treatment of gastrointestinal tumor with PM (10, 11). The application of CRS and HIPEC to CCA with PM (12, 13) has been described in a few cases with better results compared to SC in terms of overall survival.

Irreversible electroporation with BLM, called electrochemotherapy (ETC), is an innovative ablation technique for the local treatment of solid neoplasms that use the cellular membrane's electric-induced permeabilization of the neoplastic cells to increase the BLM penetrating power, introduced via the bloodstream, and his cytotoxicity (14). The application of probes around the neoplastic tissue, positioned under ultrasound (US) or computed tomography (CT)-scan guidance, delivers high-voltage electric pulses producing the formation of nanopores on the cell membrane of the neoplastic cells (15). This effect can induce by itself tumor death by apoptosis. However, it can also be used to increase the entrance of an intravenously injected drug (like BLM), which alone has a low ability to invade the intracellular space. BLM is exceptionally toxic once within the cell, but this high intrinsic cytotoxicity is limited due to BLM's inability to diffuse through the plasma membrane (14, 16–18). BLM is then intravenously injected, and, with the application of electroporation, the entrance in the neoplastic cells is 300–700 times increased with consequent apoptosis and death of the neoplastic cells (19, 20). The peculiar feature of ECT is that the effect is limited to the cell membrane, preserving the adjacent extracellular matrix structures and making this technique a useful option for tumors located next to fragile structures, like, in the case of liver tumors, major blood vessels, and major bile ducts. Accordingly, this technique was first applied to the treatment of cutaneous tumors and prostate tumors. In the last 10 years, it started to be also used to treat other neoplasia located in close contact with structures, like vessels, bile ducts, bowels, and urethra. More recently, for this highly selective effect, ECT has also been used to treat liver primary and metastatic liver tumors, including CCA (15).

The aim of reporting this case is assessing feasibility, safety, and short-term results of the simultaneous application of CRS and HIPEC with cisplatin and mitomycin and ECT with BLM, as salvage procedure for a patient with massive intrahepatic and peritoneal recurrence of CCA.

## Case Presentation

**Figure 1:**
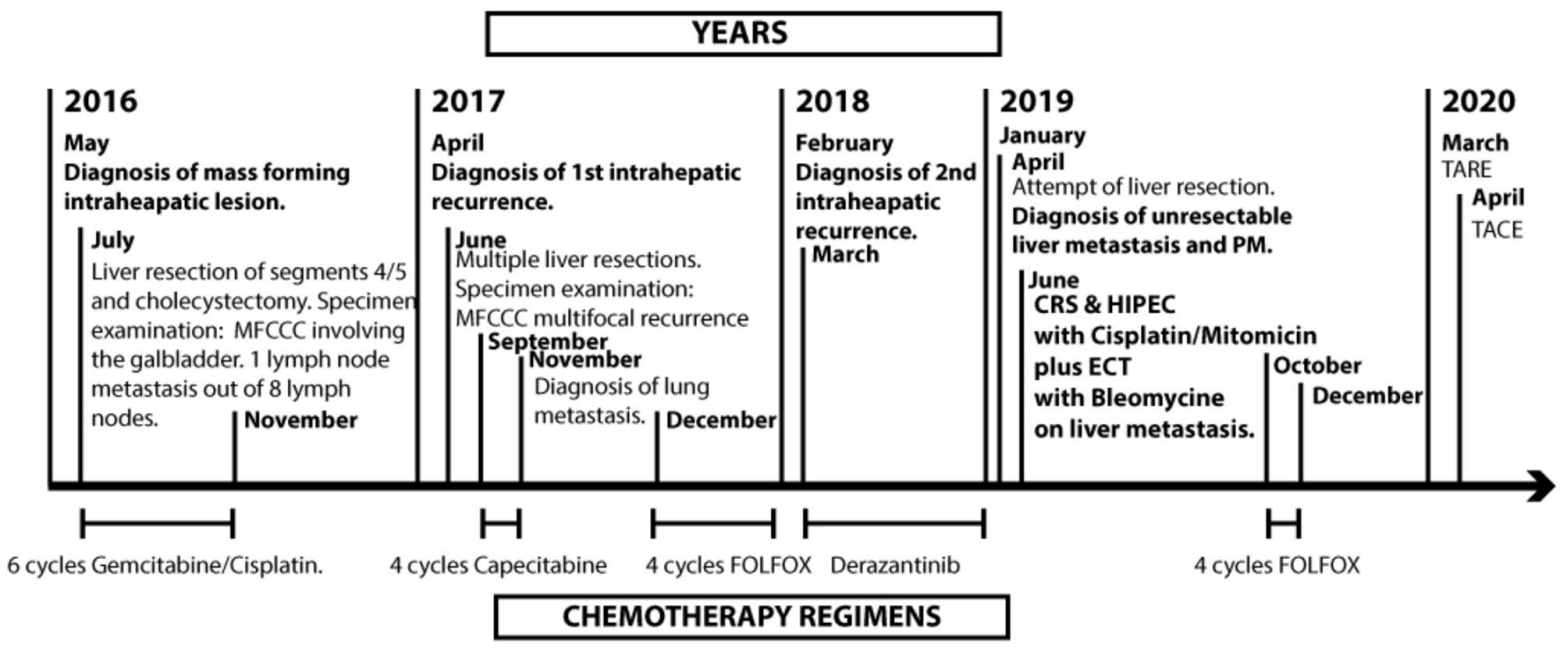
Timeline. MFCC, mass forming cholangiocarcinoma; PM, peritoneal metastasis; CRS, cytoreductive surgery; HIPEC, hyperthermic intraoperative chemotherapy; ECT, electrochemotherapy; TARE, trans-arterial radio embolization; TACE, trans-arterial chemoembolization.

We present a patient that underwent radical liver resection for intrahepatic CCA and then re-underwent radical liver resection for a first intrahepatic recurrence; nonetheless, he developed a second unresectable intrahepatic recurrence with PM and was referred to our institution where CRS, ECT with BLM, and HIPEC were performed (Figure 1).

In July 2016, an otherwise healthy 47-year-old male patient underwent another institution liver resection of segments 4 and 5 and cholecystectomy for an intrahepatic CCA; the histologic examination of the specimen revealed a “mass forming” cholangiocarcinoma with gallbladder invasion and with one positive hilar lymph node of the eight sampled. The surgical margins were clear. After the operation, the patient received six cycles of adjuvant chemotherapy with gemcitabine and cisplatin.

**Figure 2:**
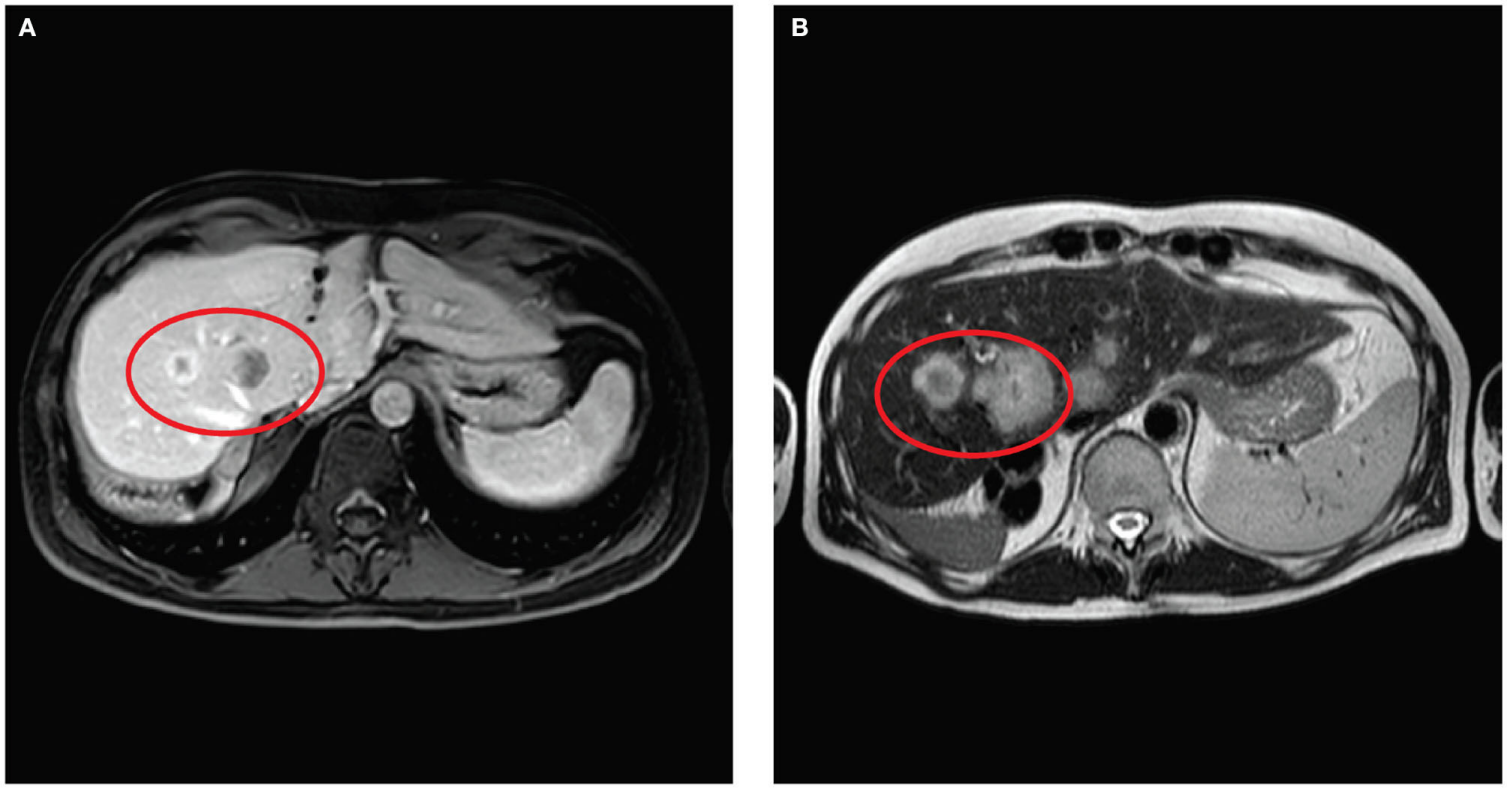
**(A)** Magnetic resonance post-contrast (gadolinium enhanced) axial MRI performed 6 months before surgery show low peripheral enhancement. **(B)** Axial MR T2 WI, performed 1 month before surgery, show slightly hyperintense lesions visible in the red circle referable to colangiocarcinoma recurrence, located at the hepatic hilum, quickly grow in size.

In June 2017, the patient developed a massive intrahepatic recurrence with ten peripheral lesions surgically resected in the same hospital with multiple hepatic wedge resections (Wedge of segments 1–6–7, Wedge of segments 4–8, Wedge of segments 5–6, and a Wedge of segment 7). The margins were clear, and no vascular invasion nor perineural invasion was observed on the surgical specimen. After surgery, the patient received four cycles of adjuvant chemotherapy with capecitabine, followed by five chemotherapy cycles with folinic acid, fluorouracil, and irinotecan (FOLFIRI). Later on, considering the tumor progression with emergence at the radiological CT scan of lung metastasis and a new liver recurrence (Figure 2A), the patient received systemic therapy with TKI Pan-FGFR Derazantinib with limited effect on tumor progression.

In the same hospital in April 2019, the patient underwent another attempt of hepatic surgical resection. However, due to the burden of the hepatic disease and the intraoperative detection of PM presence on the pelvic floor and on the lower quadrants, no demolition procedures on the liver or on the abdominal cavity PM were carried.

In July 2019, the patient was referred to our center; a restaging contrast-enhanced magnetic resonance was performed, revealing the presence of 12 hepatic metastatic lesions, the biggest one measuring 49 × 27 mm located in segment 4 (Figure 2B) and in close contact with the right portal vein, hepatic artery, and biliary duct. Peritoneal malignant nodulation was also detected on the right lobe's anterior margin (19 mm of diameter) and in the epigastric region (<10 mm of diameter).

In consideration of the rapid volumetric increase of the deepest lesion in close contact with the hepatic hilar structures, of the peritoneal involvement, and of the excellent performance status of the patient (PCOS score 0–1), the case was discussed in a multidisciplinary team meeting of surgeons, oncologists, and radiologists. It was decided to proceed with CRS and HIPEC for the peritoneal carcinomatosis and with debulking surgery and ECT with BLM on the liver to prevent the progression of the biggest metastasis located in close contact with the hilar structures.

**Figure 3:**
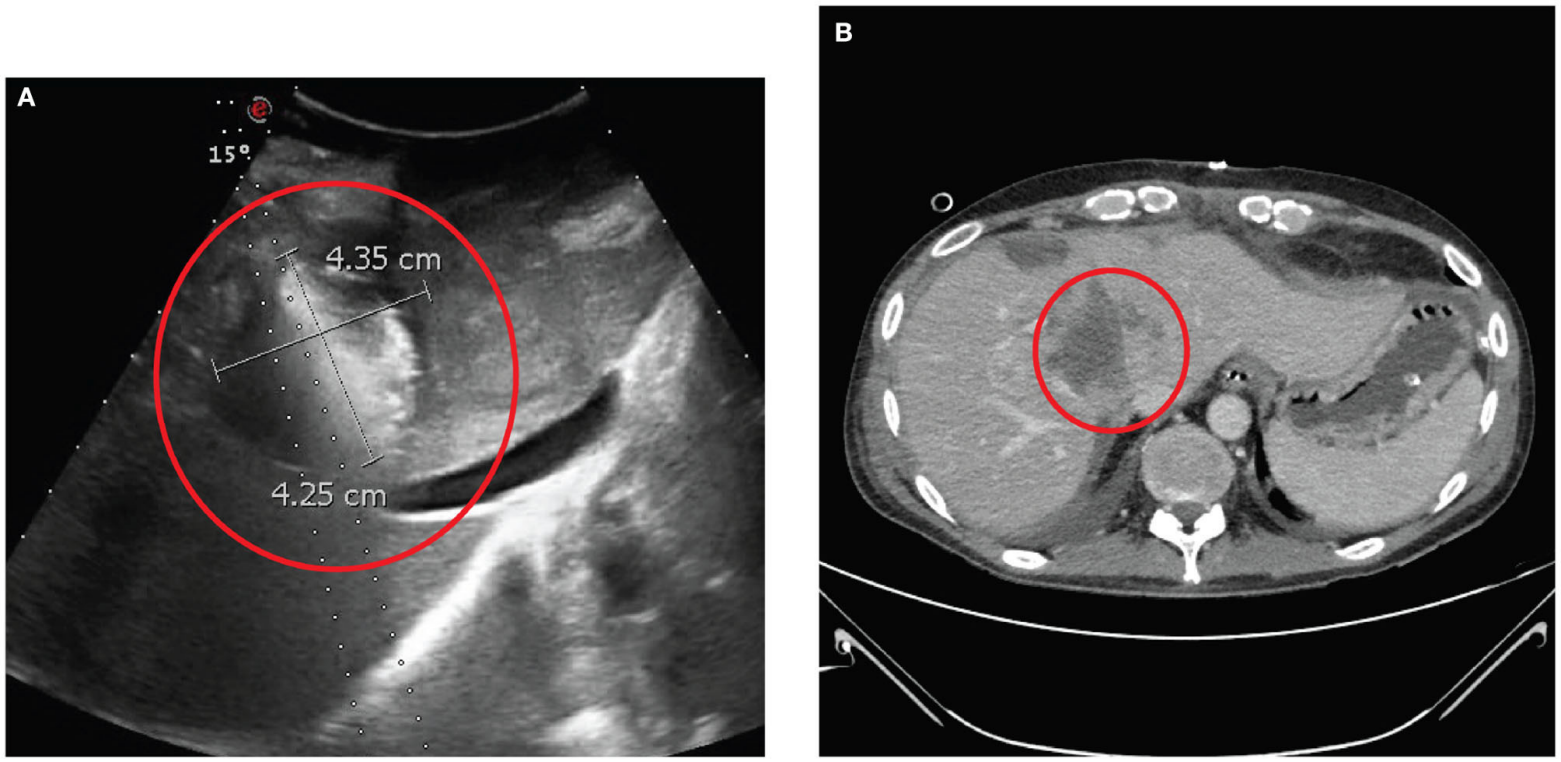
**(A)** The probes are positioned intraoperatively under ultrasound. **(B)** Contrast computer tomography scan performed after 10 days from the surgery revealing complete ipodensity of the treated area indicating full necrosis of the neoplasia induced by electrochemotherapy with bleomycine.

Hence, in July 2019, the patient underwent CRS and HIPEC and ECT with BLM. After access to the abdominal cavity, we completed a rigorous exploration. We found PM in hypogastrium, left iliac fossa, right iliac fossa, right diaphragmatic peritoneum, and greater omentum. We assessed a peritoneal carcinomatosis index (PCI) of 10. The first surgical step was the resection of two liver metastases located on segments III and IV with THUNDERBEAT (Olympus®). Then, we performed ECT with BLM on four hepatic lesions. We positioned under US guidance five electrode needles [VG-12 (1.2 mm) + 4.0 cm, IGEA S.r.l., Carpi, Italy] at the cardinal points of the metastasis located on the IVs and other four electrode needles [VG-12 (1.2 mm) + 4.0 cm, IGEA S.r.l., Carpi, Italy], at the cardinal points of the metastasis located on the II–VIIIs (Figure 3A). Then, we injected 29.4 mg of BLM. After 10 min, eight pulses of 1,000 ms at 5 kHz (Cliniporator®, IGEA S.r.l., Carpi, Italy) were recorded. The ECT procedure took 40 min. The CRS cytoreductive surgery consisted of anterior rectal resection, parietal peritonectomy, total omentectomy, appendectomy, and resection of a diaphragmatic metastasis. PM of the mesentery of the ileocecal valve and of the sigmoid mesocolon was also removed. This phase was followed by intraperitoneal hyperthermic chemotherapy with cisplatin (196 mg) and mitomycin (31.36 mg) at 41°C for 90 min (Performer HT®, RanD S.p.A., Medolla, Modena, Italy). At the end of the surgical procedure, the completeness of cytoreduction score (CC-s) 0 was obtained.

In the postoperative period, we observed acute kidney injury (RIFLE-I) (21) with maintained urine output (minimum 1.2 ml/kg/hr). We observed the creatinine peak on the fourth postoperative day, 3.03 mg/dL with eGFR 22 ml/min/1.73 mq. The patient was treated with adequate rehydration, and no hemodialysis was required. The patient also developed fever with bacteremia from Staphylococcus epidermidis requiring therapy with levofloxacin.

Eleven days after surgery, the patient was restaged with an abdominal CT scan revealing complete necrosis of the lesion with the absence of intralesional enhancement (Figure 3B). The patient returned home on the 19th postoperative day.

The patient's liver histological examination indicated two metastases from CCA, while the histological examination of the peritoneal specimens revealed six metastasis from CCA. The colonic specimen indicated the presence of neoplastic infiltration of the perivisceral tissue.

**Figure 4:**
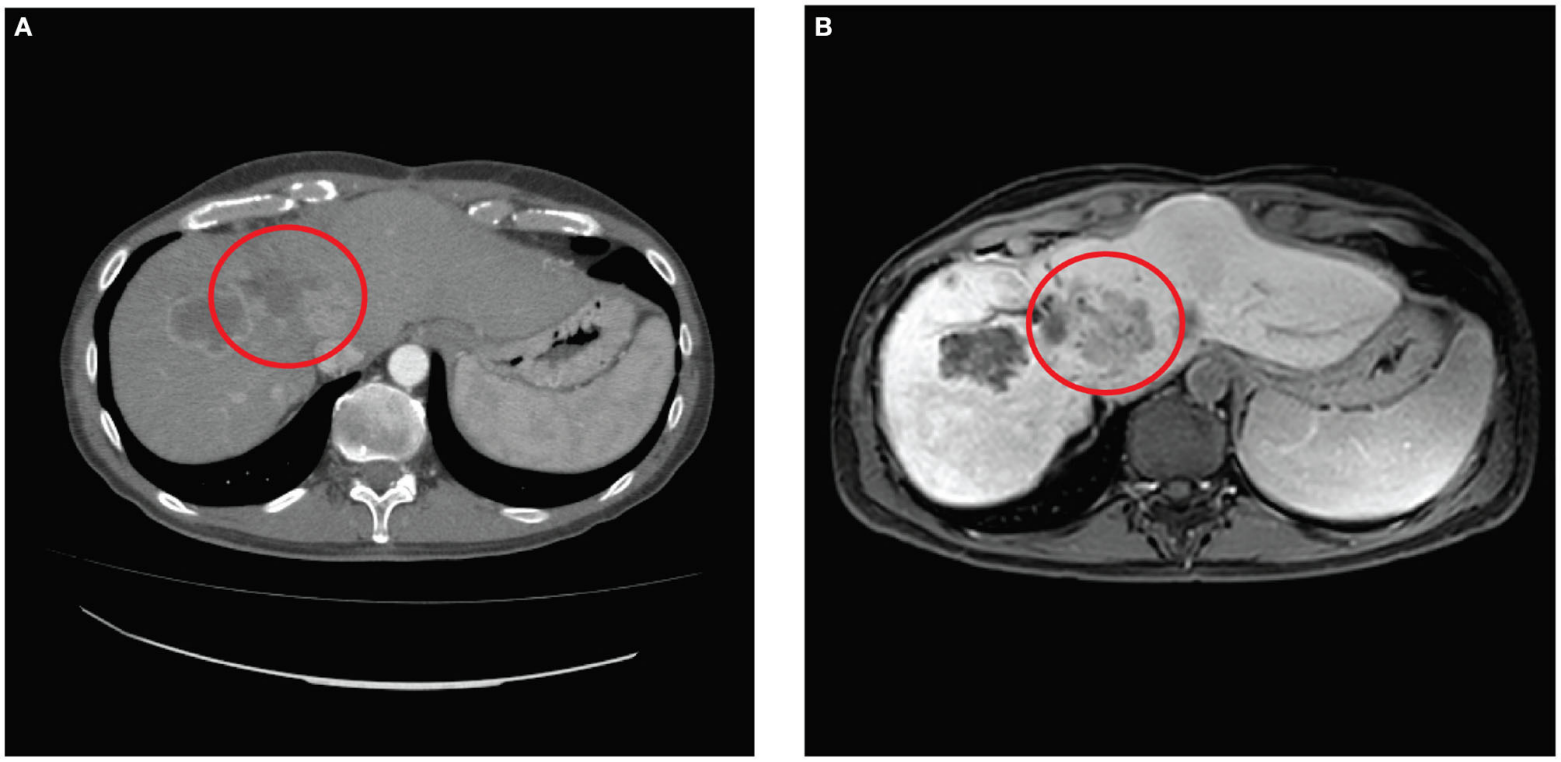
**(A)** Contrast computed tomography scan performed with contrast 6 months after surgery and **(B)** magnetic resonance scan performed with gadolinium contrast T1 WI 10 months after surgery show in both that the electrochemotherapy-treated neoplasia resulted permanently ipodense indicating the necrosis of the lesion.

In consideration of the initial stability of the progression, the patient received further four cycles of adjuvant chemotherapy with FOLFOX after 3 months from surgery and sequentially radioembolization with 1.5 Gbq of Yttrium-90 on a left liver metastasis after eight and TACE with Embocept® and 40 mg of doxorubicin on a left-lobe remnant liver metastasis after 10 months. In all the restaging CT scans performed, the liver metastasis treated with ECT appeared permanently hypodense, and no contrast enhancement was observed about the obtained necrosis (Figure 4A).

At 1 year from the treatment, the patient is in good general conditions, performance status 0; the last MR-scan revealed the liver disease's stability (Figure 4B) while the disease's lung progression was detected.

## Discussion

The aforementioned is the first case report about the feasibility and safety of the synchronous application of CRS and HIPEC and ECT with BLM for the treatment of intrahepatic and PM from CCA.

As previously stated, surgical resection is the only effective treatment for CCA. However, the incidence of recurrence after surgical resection is high, with more than half of the patients developing it within a year, resulting in a far worse survival prognosis. The most frequent sites of recurrence are the liver, peritoneum, and lungs. At the moment, surgery, when feasible, remains the most efficient therapy for the recurrence of CCA in the presence of either intrahepatic or peritoneal recurrence. Some recent retrospective studies (22, 23) revealed an improvement of the overall survival in patients that underwent radical surgical resection of the recurrence compared to the patient that did only chemoradiotherapy. Recently, Amblard reported the first study about the role of CRS and HIPEC for the treatment of patients with PM from CCA with promising results, in terms of overall survival, when compared to systemic chemotherapy only in the group of patients, where CC-s 0 was obtained. In this retrospective multicentric study on CRS and HIPEC on patients with PM from CCA, but without intrahepatic recurrence, the overall survival was significantly better for the CRS and HIPEC group (21.4 vs. 9.3 months). However, no difference in the progression-free survival time was observed (12). In the last years, also Pressurized Intra Peritoneal Aerosol Chemotherapy (PIPAC) is arising as a possible therapy in patients with extended PM from a wide variety of malignancies (24). PIPAC procedure, however, remains contraindicated due to the high incidence of postoperative complications when performed together with intestinal resections or other major surgical procedures (24, 25). In the explorative laparotomy before the surgical procedure reported in this publication, PM on the pelvic floor and the lower quadrants were detected. Hence, the CRS would have certainly implied an intestinal resection to obtain the CCI-0. In this contest, we chose to perform HIPEC instead of PIPAC.

Radiofrequency (RF) ablation has given good results for the treatment of intrahepatic tumor (26). This technique has been applied initially for the treatment of HCC. However, the application to perihilar and deeply located neoplasms is partially contraindicated due to the high probability of injuries that high temperatures can produce on the hepatic hilum's main structures (27). In recent years, ECT with BLM has started to be applied to unresectable liver neoplasms, mostly HCC, colorectal liver metastasis, and CCA, with good results firstly in terms of radiological response to the treatment (15). Unlike RF, this technique can be applied to deeply located lesions because it does not use thermal energy to induce neoplastic cells' necrosis. The electrical pulse energy enhances cell membrane permeability. It can be used either alone by inducing cell death with high voltage delivery or in combination with poorly permeant chemotherapy agents intravenously injected in order to increase their entrance in the tumor cells. In combination with electroporation, the intracellular concentration and therefore the cytotoxicity of BLM increase 300–700-folds, but not destroy nor modify the tissues' stromal architecture among the target lesion with lowered damages on normal cells. As a result, ECT can be applied for liver tumors in close contact with main hilar structures.

The hereby described patient was referred to our center after liver resection for a mass forming CCA and second liver resection for the primary tumor recurrence; in both operations, R0 was achieved. During the resection of the primary tumor, one of the eight lymph nodes resulted positive for tumor invasion. The second recurrence, arisen 2 years after the first operation, was unresectable due to the burden of liver involvement and too high potential risk of post-hepatectomy liver failure and for the presence of PM.

This therapeutic approach was designed (also by the patient's conscious choice) to give the patient a treatment that could give him the best of survival chances, not concerning the full oncological radicality's achievement. The PM was surgically approachable with CRS and HIPEC, and the treatment aimed to obtain a CC-s 0. On the other hand, the liver disease was unresectable with curative intent, but to give the patient the chance of other oncologic therapies was mandatory to treat the metastasis in rapid growth and close contact of hilar structures like the hepatic artery, portal vein, and hepatic vein. The fast-growing tumor had a high risk of macrovascular invasion, which would be a relative contraindication for locoregional treatments, like transarterial chemoembolization (TACE) or transarterial radioembolization (TARE). The tumor growing in the hepatic hilum had a high risk of invasion of the biliary tract, which would have resulted in obstructive jaundice, which was a contraindication not only for treatments like TACE and TARE but also for systemic chemotherapy. In the situation of unresectable CCA, the prognosis is poor, and the survival is <1 year without treatment and 11.7 months with systemic therapy (5), but approaches like TACE or TARE had quite optimistic results in terms of overall survival increase, respectively, 12.4 and 13.9 months (6, 28). Furthermore, hepatic intra-arterial and systemic chemotherapy, followed by maintenance therapy with gemcitabine, had brought comparable results in terms of overall survival. On the other side, the technique gave excellent results in patients downstaged and directed to liver resection or thermoablation (29).

The feasibility of the procedure shows that this strategy is applicable when PM that can be treated with CRS and HIPEC is associated with synchronous liver metastasis that is unresectable due to their multiplicity, a particular location, or a too high risk of postoperative complications if a major hepatectomy is associated with a CRS and HIPEC procedure. In this case, ECT with BLM can be considered the “bridge” therapy to allow the patient to continue the most effective therapies in terms of overall survival (6). The rationale of the two procedures' combination and synchronous application is based on the reasoning that the chemotherapeutic agents carry out their actions in two different spaces. Actually, on the one hand, BLM works in the systemic bloodstream (30) and then in the liver through the high intracellular concentration induced by electroporation; meanwhile, on the other hand, mitomycin and cisplatin essentially on the peritoneal surface, as the plasma-peritoneal barrier, prevents their significant passage into the plasma (31, 32). The safeness of the synchronous application of both these procedures, even if high doses of chemotherapy in a tiny amount of time are administered in the plasma and peritoneal spaces, is warranted by the fact that these two spaces are kept “watertight” by the plasma-peritoneal barrier, formed by peritoneal mesothelium, subserosal interstitium, and capillary walls. This barrier causes a high ratio of some cytotoxic drugs, like cisplatin and mitomycin, and a concentration between the peritoneal cavity and the plasma, maximizing the intraperitoneal anti-tumoral toxicity and minimizing the systemic toxicity of the chemotherapy (33, 34). On the other side, BLM's effective cytotoxic dose is significantly lower in ECT than the dose used in standard chemotherapeutic regimens. The permeability of the cellular membranes to the BLM is increased by electroporation (35).

This study has two significant limitations. The first is because it is a case report. Secondly is our experience in a very complex clinical case where many therapies had already been applied complicating the scenario. Other experiences are necessary in very extreme cases of primary or recurrent CCA with PM's contemporary presence. However, the synchronous application of CRS and HIPEC and ECT with BLM has been shown feasible and safe in our case, allowing that this simultaneous application may give a new treatment opportunity for patients with different tumors and synchronous PM and liver metastasis, in order to increase the overall survival. In this particular case, where the oncological radicality was impossible to obtain, the therapeutic strategy's target was to identify an effective treatment to give the patient better chances to be subjected to multidisciplinary and multidimensional treatments, obtaining the most prolonged possible survival. The therapy of choice was to treat firstly peritoneal recurrence with CRS and HIPEC to obtain a CC-s 0, which is, as reported by Amblard, linked to better overall survival. The second aim of the treatment was to treat the hilar metastasis in rapid growth in order to avoid vascular and biliary major invasion to prevent the biliary occlusion, which is a contraindication for therapies like chemoembolization or radioembolization, which at the moment are the only that can give an improvement in the overall survival.

## Conclusion

This case report indicates that CRS and HIPEC for PM from CCA combined with ECT with BLM can be a feasible and safe procedure for a patient developing intraparenchymal unresectable disease associated with a simultaneous PM. Even with the disease in progression, this patient is alive with PCOS 0–1 at 4 years from the first surgery for CCA and 3 years from liver resection for the first recurrence, which has to be considered an outstanding result for such aggressive neoplasm.

## Data Availability Statement

The raw data supporting the conclusions of this article will be made available by the authors, without undue reservation.

## Ethics Statement

Ethical review and approval was not required for the study on human participants in accordance with the local legislation and institutional requirements. The patients/participants provided their written informed consent to participate in this study. Written informed consent was obtained from the individual(s) for the publication of any potentially identifiable images or data included in this article.

## Author Contributions

MS performed the surgery, designed the study, and wrote the article. EP collected the data and wrote the article. PF, FC, MT, VA, and EG designed the study and revised the article. BB collected the data and revised the article. MB performed the surgery, designed the study and revised the article. CM collected the data. AP performed the surgery and collected the data. LA performed the surgery, designed the study wrote the article and revised the article. All authors contributed to the article and approved the submitted version.

## Conflict of Interest

The authors declare that the research was conducted in the absence of any commercial or financial relationships that could be construed as a potential conflict of interest.
